# Association of MTHFR and DNMT-1 Gene Polymorphisms with Acute Coronary Syndrome in Patients Admitted to the Emergency Department

**DOI:** 10.3390/jcm14082767

**Published:** 2025-04-17

**Authors:** Fulya Yukcu, Murtaza Kaya, Raziye Akcilar, Fatmagul Can, Harun Yildirim

**Affiliations:** 1 Department of Biophysics, Faculty of Medicine, Kutahya Health Sciences University, Kutahya 43100, Turkey; 2 Department of Emergency Medicine, Faculty of Medicine, Kutahya Health Sciences University, Kutahya 43100, Turkey; murtaza.kaya@ksbu.edu.tr (M.K.); harun.yildirim@ksbu.edu.tr (H.Y.); 3 Department of Physiology, Faculty of Medicine, Kutahya Health Sciences University, Kutahya 43100, Turkey; raziye.akcilar@ksbu.edu.tr; 4 Department of Medical Biochemistry, Faculty of Medicine, Kutahya Health Sciences University, Kutahya 43100, Turkey; fatmagul.can@ksbu.edu.tr

**Keywords:** acute coronary syndrome, MTHFR, DNMT1 protein, genetic polymorphism, PCR, RFLP

## Abstract

**Background/Objectives:** Acute coronary syndrome (ACS) is a critical cardiovascular condition influenced by genetic and environmental factors. Polymorphisms in methylenetetrahydrofolate reductase (MTHFR) and deoxyribonucleic acid methyltransferase-1 (DNMT-1) genes are linked to cardiovascular diseases, yet their specific roles in ACS pathogenesis remain unclear. This study examines the association of MTHFR C677T and DNMT-1 +32204 A/G polymorphisms with ACS and their potential contribution to genetic risk profiling. **Methods:** A case–control study was conducted with 212 participants, including 106 ACS patients and 106 controls. Peripheral blood samples were collected and analyzed to determine genotypic and allelic frequencies using the polymerase chain reaction–restriction fragment length polymorphism (PCR-RFLP) technique. Statistical analyses were performed to assess associations between gene polymorphisms and ACS risk. **Results**: The MTHFR C677T polymorphism showed a strong association with ACS. The CC genotype significantly increased risk (OR: 7.34; 95% CI: 2.28–23.6; *p* < 0.001), while the C allele was also associated with higher susceptibility (OR: 2.21; 95% CI: 1.46–3.35; *p* < 0.001). Conversely, the T allele exhibited a protective effect, being more frequent in controls (62.9% vs. 37.1% in ACS; *p* = 0.000). Elevated troponin I levels in ACS patients with the TT genotype (*p* = 0.025) suggested a link between MTHFR variants and disease severity. However, DNMT-1 +32204 A/G polymorphisms showed no significant association with ACS risk. **Conclusions:** The MTHFR C677T polymorphism influences ACS susceptibility, with the CC genotype as a risk factor and the T allele offering potential protection.

## 1. Introduction

Sudden cardiac death (SCD) is a leading cause of mortality (15–20%), with cardiovascular diseases accounting for many of these deaths [1]. Coronary artery disease (CAD) remains the primary pathology associated with SCD; however, advancements in therapeutic approaches over the past decade have significantly reduced mortality rates due to CAD. The genetic contribution to SCD susceptibility can be categorized into genetic variants associated with known inherited cardiac disorders and those contributing to SCD susceptibility without being linked to a known cardiac disorder or electrocardiogram (ECG) pattern [2].

The development of acute coronary syndrome (ACS) results from a complex interaction of genetic and environmental factors [3]. Emerging evidence suggests that epigenetics represents a rapidly advancing field that could offer solutions to the persistent clinical burden of cardiovascular diseases [4]. However, the genetic factors and underlying mechanisms involved in the pathogenesis of ACS remain incompletely understood.

Methylenetetrahydrofolate reductase (MTHFR) is a coenzyme of vitamin-B12-dependent methionine synthase, which facilitates the conversion of homocysteine to methionine [5,6]. The MTHFR enzyme plays a critical role in the folate cycle and significantly contributes to homocysteine amino acid metabolism [7]. Various mutations that reduce the enzyme’s function have been reported in the MTHFR gene, the most common of which is the C677T mutation, leading to elevated homocysteine levels [8]. Elevated homocysteine levels have been shown to be associated with an increased risk of cardiovascular, cerebrovascular, and peripheral arterial diseases [9,10].

Deoxyribonucleic acid (DNA) methylation occurs through the addition of a methyl group to CpG (C, cytosine; p, phosphate; G, guanine) regions by the DNA methyltransferase enzyme. The enzyme responsible for adding methyl groups to CpG regions in DNA is known as DNA methyltransferase (DNMT). In mammals, three DNMTs have been identified: DNMT-1, DNMT-3A, and DNMT-3B [11]. DNMT-1 is an enzyme involved in this reaction, responsible for maintaining epigenetic marks, thereby ensuring their replication during each cell division [4].

Several documented observations have shown a correlation between increased DNMT-1 levels, decreased peroxisome proliferator-activated receptor gamma levels, and elevated pro-inflammatory cytokines in macrophages of apolipoprotein E-deficient mice fed an atherogenic diet. This correlation has also been observed in patients with atherosclerosis [12]. Numerous studies have linked changes in DNA methylation to the development of cardiovascular diseases [13,14].

The aim of this study is to investigate an association between clinical findings and the MTHFR C677T and DNMT-1 +32204 A/G gene polymorphisms among patients with ACS, and to determine whether genotypic variations play a role in the development and pathophysiology of the disease. Additionally, the study aims to provide insights for future research that may contribute to slowing disease progression.

## 2. Materials and Methods

### 2.1. Study Design

This prospective cross-sectional study was conducted at the Department of Emergency Medicine, Faculty of Medicine, Kutahya Health Sciences University, and the Emergency Department of Kutahya City Hospital between 22 April 2024 and 22 October 2024. Blood samples were collected from participants and analyzed at the Physiology Laboratory of Kutahya Health Sciences University.

### 2.2. Patient Selection

The study included patients over the age of 18 years who presented to the emergency department with chest pain. Inclusion also required the presence of electrocardiographic changes and alterations in myocardial enzyme levels, confirming the acute cardiac event. Exclusion criteria ruled out patients below the age of 18 years diagnosed with malignancies, coagulation disorders, myocarditis, or endocarditis, as well as individuals with arrhythmias or pregnancy. Diagnoses of ACS were determined based on the criteria established by the American College of Cardiology (ACC)/American Heart Association (AHA) and the European Society of Cardiology (ESC), encompassing unstable angina pectoris, non-ST elevation myocardial infarction (NSTEMI), and ST-elevation myocardial infarction (STEMI) [15,16]. Ischemic changes on an ECG may include ST-segment depression, T-wave inversion, or transient ST-segment elevation, particularly in contiguous leads. For a diagnosis of STEMI, ST-segment elevation (STE) must be present in at least two contiguous leads. V2-V3 leads: women: ≥1.5 mm, men ≥ 40 years: ≥2 mm, and men < 40 years: ≥2.5 mm; other leads: STE ≥ 1 mm; V7-V9 leads (posterior STEMI): STE ≥ 0.5 mm; V3R/V4R leads (right-sided): STE ≥ 1 mm (≥0.5 mm for individuals under 30 years old). For the control group, participants included individuals over the age of 18 years who experienced chest pain but were not diagnosed with ACS, along with healthy volunteers who had no personal or familial history of ACS or atherosclerotic heart disease. 

### 2.3. Data Collection

Demographic characteristics such as the sex and age of the patients included in the study were recorded. Furthermore, routine biochemical parameters commonly assessed in patients presenting to the emergency department were collected. These included troponin I, sodium, potassium, urea, creatinine, glucose, aspartate aminotransferase (AST), and alanine aminotransferase (ALT). Peripheral blood samples collected from the patients were drawn into sterile vacuum tubes containing approximately 2 mL of ethylenediaminetetraacetic acid (EDTA). The collected samples were stored at −20 °C until the DNA isolation process was performed.

### 2.4. DNA Isolation and PCR-RFLP Methods

DNA was extracted from EDTA–containing blood samples using a Monarch Genomic DNA Purification Kit (New England Biolabs, NEB, Ipswich, MA, USA). The MTHFR C677T (rs1801133) and DNMT-1 +32204 A/G (rs2228611) gene polymorphisms were analyzed using PCR and RFLP methods [17,18]. Details regarding the primer sequences, PCR conditions, restriction enzymes, and genotyping results are summarized in Table 1. The product sizes for the MTHFR C677T gene polymorphism were identified as 198 bp for CC, 175 bp for TT, and 175-198 bp for CT. Similarly, for the DNMT-1 +32204 A/G gene polymorphism, the product sizes were determined as 329 bp for AA, 143-186-329 bp for GA, and 143–186 bp for GG. 

### 2.5. Sample Size

**Figure 1:**
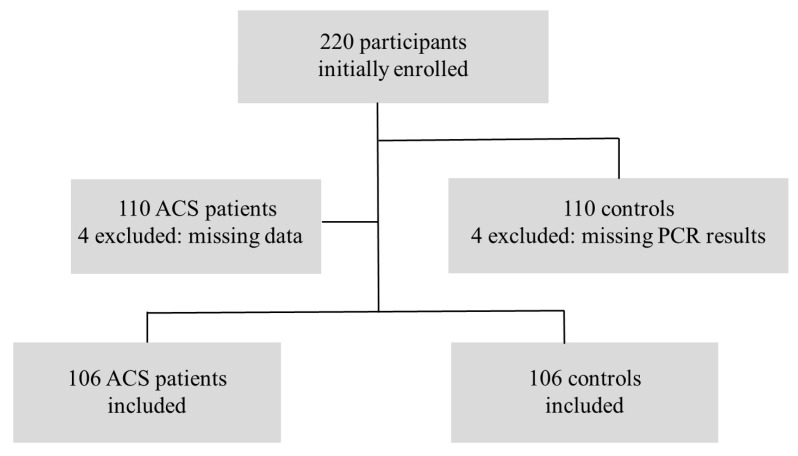
Flowchart of the study participants.

The sample size and power calculation were based on the odds ratio (OR) of 2.06 reported in a study by Feng AL et al. [17]. Utilizing G-power 3.1 software, with a medium effect size of 0.25, an error margin of 0.05, and a confidence level of 0.95, the necessary sample size was determined to be 110 participants per group. This calculation was conducted to achieve a power value of 0.80 for detecting differences between two independent groups. Initially, a total of 220 participants were enrolled in the study, comprising 110 ACS patients and 110 controls. Subsequently, four patients from the patient group and four controls from the control group were excluded due to missing data and PCR results, respectively. Consequently, 212 participants (106 ACS patients and 106 controls) were included in the final analysis. Flowcharts are presented in Figure 1.

### 2.6. Statistical Analysis

Statistical analyses were performed using the Statistical Package for Social Sciences (SPSS) software version 27.0.1 (IBM Corp., Armonk, NY, USA). Continuous variables were tested for normal distribution using the Kolmogorov–Smirnov test. Normally distributed variables were expressed as mean ± standard deviation (SD) and analyzed using parametric tests, such as the Student’s *t*-test and one-way analysis of variance (ANOVA). Non-normally distributed variables were analyzed using non-parametric tests where applicable. Categorical variables were expressed as numbers and percentages and compared using a chi-square (χ^2^) test. Demographic and clinical variables between groups were compared using independent samples t-tests or χ^2^ tests. Genotype and allele distributions were analyzed for associations with ACS using χ^2^ tests. The relationships between genotypes and clinical characteristics within the ACS and control groups were evaluated using ANOVA and χ^2^ tests as appropriate. Hardy–Weinberg equilibrium was assessed for genotype distributions within both the ACS and control groups using a chi-square test. OR and 95% confidence intervals (CI) were calculated to evaluate the associations between genotypes, alleles, and ACS risk. A *p*-value of less than 0.05 was considered statistically significant for all tests.

## 3. Results

Genotype distributions between both controls and ACS patients were in accordance with the Hardy–Weinberg equilibrium (*p* > 0.05). The demographic and clinical characteristics of the study population are summarized in Table 2. The study included 106 patients diagnosed with ACS and 106 control individuals. The mean age of the ACS group was significantly higher than the control group (61.5 ± 14.1 years vs. 51.0 ± 19.1 years; *p* < 0.001). There were no significant differences in sex distribution between the groups (*p* = 0.778). Biochemical parameters such as glucose, potassium, and AST levels were significantly elevated in the ACS group compared to controls (*p* < 0.001, *p* = 0.004, and *p* = 0.003, respectively). Troponin I levels > 100 ng/L—the institutional reference threshold for indicating marked myocardial injury—were observed in 82.2% of ACS patients, whereas only 17.8% of controls had such levels (*p* < 0.001). The 18 patients in the control group with elevated troponin I levels had non-cardiac conditions, such as infections, renal failure, ischemic stroke, and gastrointestinal bleeding.

Table 3 shows the distribution of MTHFR C677T and DNMT-1 +32204 A/G genotypes and allele frequencies between the control and ACS groups. For the MTHFR C677T polymorphism, the CC genotype was significantly more frequent in the ACS group compared to the control group (*p* < 0.001), with an OR of 7.34 (95% CI: 2.28–23.6). The CT genotype was also significantly associated with the ACS group (*p* = 0.03, OR: 3.42, 95% CI: 1.07–10.8), while the TT genotype was used as the reference. Similarly, the C allele frequency was higher in the ACS group than in the control group (*p* < 0.001, OR: 2.21, 95% CI: 1.46–3.35). In contrast, for the DNMT-1 +32204 A/G polymorphism, no significant differences were observed in the genotype or allele distributions between the two groups (*p* > 0.05). The GG genotype served as the reference for comparisons, and the analysis revealed no significant associations for the GA or AA genotypes, nor for the A allele.

Table 4 explores the relationship between MTHFR C677T and DNMT-1 +32204 A/G genotypes and the clinical characteristics of the control group. No statistically significant associations were found between MTHFR C677T genotypes (CC, CT, and TT) and the evaluated variables, including age, sex, glucose, BUN, creatinine, electrolytes, liver enzymes (ALT and AST), or troponin I levels (*p* > 0.05). Similarly, no significant differences were observed between DNMT-1 +32204 A/G genotypes (GG, GA, and AA) and these characteristics (*p* > 0.05). Thus, the findings indicate no genotype-related differences in the control group’s demographic or clinical parameters.

Table 5 investigates the relationship between MTHFR C677T and DNMT-1 +32204 A/G genotypes and clinical characteristics in the ACS group. For the MTHFR C677T polymorphism, a significant difference was observed in troponin I levels across genotypes, with the TT genotype showed the highest percentage of individuals with troponin I > 100 ng/L (*p* = 0.025). However, no significant associations were identified for other clinical variables, such as age, sex, glucose, or liver enzyme levels (*p* > 0.05 for all). Regarding the DNMT-1 +32204 A/G polymorphism, no statistically significant differences were observed in any of the evaluated characteristics, including troponin I levels, across genotypes (*p* > 0.05 for all). Similarly, no significant associations were found between genotypes and the distribution of ACS diagnostic categories, such as STEMI, NSTEMI, or unstable angina.

**Figure 2:**
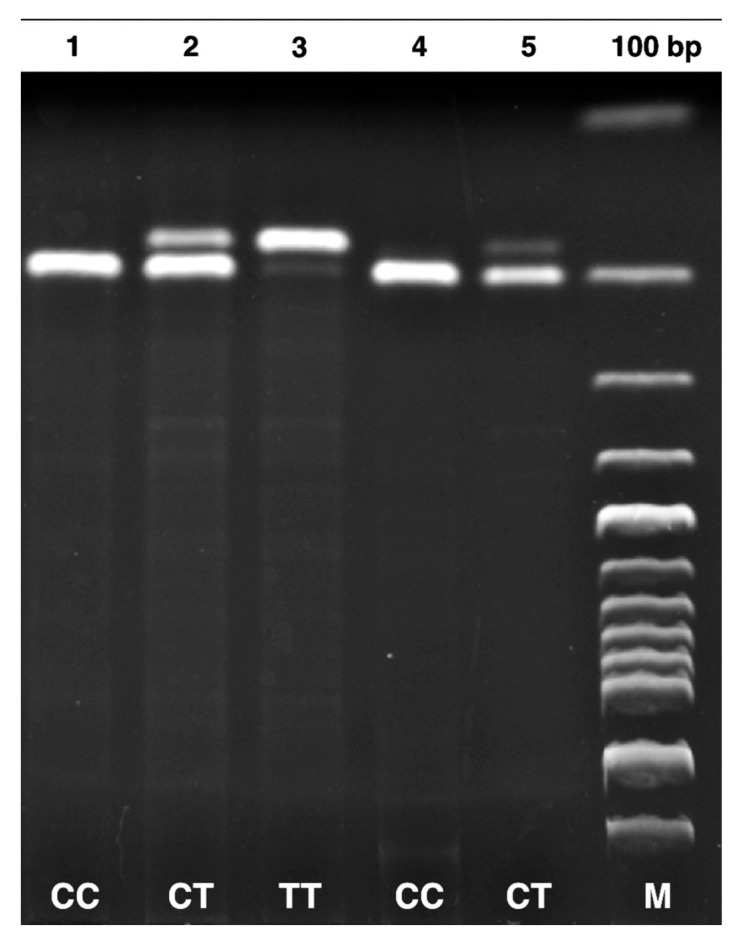
Gel electrophoresis of PCR–amplified product of MTHFR C677T gene polymorphism. CC (198 bp), TT (175 bp), CT (175–198 bp), and M (100 bp DNA molecular weight marker (abm, Katalog No: G193)).

**Figure 3:**
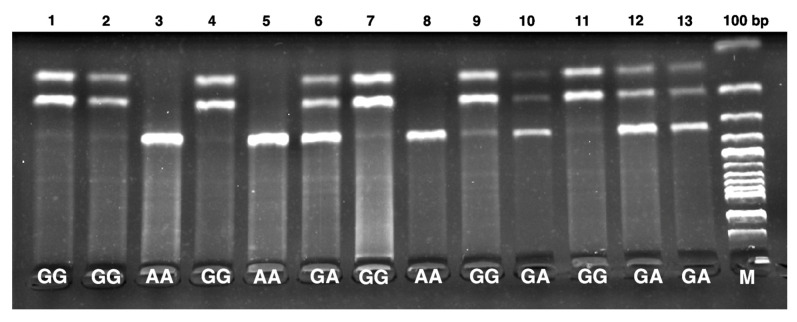
Gel electrophoresis of PCR–amplified product of DNMT-1 +32204 A/G gene polymorphism. AA (329 bp), GA (143–186–329 bp), GG (143–186 bp), and M (100 bp DNA molecular weight marker (abm, Katalog No: G193)).

Figure 2 and Figure 3 show patient and control truncation products for the MTHFR C677T and DNMT-1 +32204 A/G gene polymorphisms, run on a 2% agarose gel, and viewed under ultraviolet light.

## 4. Discussion

The effects of genetic factors on ACS have been increasingly elucidated in recent studies. It is believed that genetic variations, together with epigenetic mechanisms, contribute to the pathogenesis of ACS by influencing pro-inflammatory processes and the stability of atherosclerotic plaques. This study investigated the effects of MTHFR C677T and DNMT-1 +32204 A/G gene polymorphisms on ACS. Our findings suggest that while some genotypes may increase the risk of ACS, others may play a protective role.

In a study conducted by Miao-Nan Li et al., the mean age of 310 patients diagnosed with ACS was reported as 62.5 ± 10.8 years, with a sex distribution of 57.7% male (179 individuals) and 42.3% female (131 individuals) [19]. Similarly, in our study, the mean age of the ACS group was 61.5 ± 14.1 years, with 51.1% male (67 individuals) and 48.1% female (39 individuals). Both studies highlight that ACS is more commonly observed in males; however, the sex distribution in our study was more balanced. Moreover, the similarity in mean age further supports the observation that ACS is more prevalent in older populations. 

In comparison to the genotypic distribution reported by Miao-Nan Li et al., notable differences were observed in our study population. While their analysis of Chinese ACS patients showed a CC genotype frequency of 25.2% and a T allele frequency of 52.7%, our findings indicated a markedly higher prevalence of the CC genotype (49.1%) and a lower frequency of the T allele (38.2%) [19]. These discrepancies may reflect population-specific genetic variability as our study was conducted in a different ethnic and geographical context. Such differences highlight the importance of considering genetic background when evaluating the association between MTHFR polymorphisms and cardiovascular risk.

In our study, the MTHFR C677T polymorphism showed a strong association with ACS, with the CC genotype being particularly prominent among patients (OR: 7.34; 95% CI: 2.28–23.6). Individuals with the CC genotype were found to have over seven times greater odds of developing ACS compared to those with the TT genotype. These findings are in line with a meta-analysis conducted by Lakkakula et al., which demonstrated that MTHFR 677C>T mutant genotypes were significantly associated with an increased risk of vascular complications in patients with sickle cell disease [20]. Although the patient populations and clinical conditions differ, both studies support the notion that MTHFR polymorphisms may influence vascular disease processes, possibly through homocysteine-related endothelial dysfunction. The consistency between our findings and those in the broader literature underscores the relevance of MTHFR C677T as a potential genetic marker in cardiovascular risk assessment.

MTHFR gene polymorphisms, particularly the C677T variant, have a significant impact on plasma homocysteine levels. This genetic variant reduces MTHFR enzyme activity, leading to impaired homocysteine metabolism and consequently to hyperhomocysteinemia. Homocysteine has been associated with endothelial dysfunction, oxidative stress, and pro-inflammatory states, and is recognized as an independent risk factor for cardiovascular disease. Therefore, the potential cardiovascular effects of MTHFR polymorphisms may be mediated through alterations in homocysteine levels [21,22]. Although homocysteine levels were not measured in our study, evaluating genotype variations in more detail along with assessing biomarkers like homocysteine may help clarify the potential effects of MTHFR and its interaction with environmental factors.

A study conducted in a Hakka population in southern China investigated the association of the MTHFR C677T polymorphism with ACS but found no significant relationship between the genotypes and ACS risk [23]. This contrasts with our findings, where the MTHFR CC genotype was identified as a significant risk factor for ACS. The discrepancy between the two studies may be attributed to genetic diversity and environmental factors unique to each population. 

In our study, the ACS group was observed to be significantly older than the control group, with markedly elevated troponin I levels (troponin I > 100 ng/L: 82.2% vs. 17.8%; *p* < 0.001). This difference in troponin I levels is widely recognized in the literature as a prominent biochemical marker of ACS [19]. Notably, an increase in troponin I levels was observed in patients with the MTHFR TT genotype (*p* = 0.025). This finding aligns with the results of Huipu Xu’s study, which linked the TT genotype to a higher plaque burden and instability [24]. However, our study did not include a specific evaluation of plaque instability or characteristics.

In a study by Chunyan Peng et al., conducted in a Chinese Han population, the DNMT-1 rs2228611 polymorphism was reported to be significantly associated with coronary artery disease (CAD), with the A allele showing a protective effect (OR = 0.404; 95% CI: 0.184–0.884; *p* = 0.023) [23]. In contrast, our study did not find a significant association between DNMT-1 rs2228611 genotypes and ACS. Additionally, we observed no significant differences in clinical parameters such as age, glucose, creatinine, or troponin I levels across DNMT-1 genotypes in the ACS group. While a study by Peng et al. emphasizes the potential role of DNMT-1 variants in CAD pathogenesis via epigenetic regulation, our findings suggest that such effects may not be universally observed across all cardiovascular conditions or populations. Chen HL et al. reported that the AA genotype of DNMT-1 rs2228611 was associated with an increased risk of essential hypertension in males, suggesting a possible role in cardiovascular pathophysiology [25]. However, considering the scarcity of studies evaluating DNMT-1 polymorphisms specifically in ACS populations, further research is warranted to elucidate their relevance in this clinical context. 

Our study has a few limitations that should be noted. While the sample size was adequate for our analyses, larger and more diverse populations would provide stronger generalizability of the findings. Additionally, we did not measure serum homocysteine levels, which could have offered more insight into the functional implications of MTHFR polymorphisms, and we lacked data on potential confounders, such as lifestyle factors, dietary folate intake, or pharmaceutical usage. Furthermore, the study did not explore detailed plaque characteristics through imaging, which might have provided a clearer understanding of the relationship between genotypes and atherosclerotic plaque behavior. The ACS group in our study was older than the control group, which may act as a potential confounding factor in cardiovascular research; however, it is worth noting that the participants were not of advanced age overall. Lastly, we did not extensively evaluate environmental factors, such as diet and lifestyle, that could influence gene–environment interactions.

## 5. Conclusions

This study demonstrates the significant role of the MTHFR C677T polymorphism in ACS. The CC genotype and C allele were identified as significant risk factors, while the T allele showed a potential protective effect. These findings suggest that genetic variations may influence ACS susceptibility and contribute to our understanding of its underlying pathophysiology.

Future studies incorporating larger cohorts and functional biomarkers such as gene–environment interactions will be important to validate these associations and further elucidate their clinical relevance.

## Figures and Tables

**Table 1:** Summary of conditions for the MTHFR 677 C/T and DNMT-1 +32204 A/G genetic analyses.

SNP	Prime Sequence (5′-3′)	Tm (°C)	PCR Product Size	Restriction Enzyme	Genotyping (bp)
**MTHFR** **677 C/T** **(rs1801133)**	**F**-TGAAGGAGAAGGTGTCTGCGGGA **R**-AGGACGGTGCGGTGAGAGTG	61 °C	198 bp	HinfI	CC: 198 CT: 175–198 TT: 175
**DNMT-1 +32204 A/G** **(rs2228611)**	**F-**CTGATCTGAAGTCTGCACGAAG **R-**TCCAGGCTCGTCTCAAACTC	58 °C	329 bp	Alw26I (BsmAI)	GG: 143–186 GA: 143–186–329 AA: 329

MTHFR: methylenetetrahydrofolate reductase, DNMT-1: DNA methyltransferase 1, SNP: single nucleotide polymorphism, Tm: primer annealing temperature, bp: base pairs, F: Forward, R: Reverse.

**Table 2:** Demographic and clinical characteristics of the groups.

Characteristic	Groups		*p*-Value
	Control (n = 106)	ACS (n = 106)	
**Age**	51.0 ± 19.1	61.5 ± 14.1	<0.001 *
**Sex**			
Female [n (%)]	42 (51.9)	39 (48.1)	0.778
Male [n (%)]	64 (48.9)	67 (51.1)	
**Glucose (mg/dL)**	122.1 ± 62.6	158.1 ± 76.8	<0.001 *
**BUN (mg/dL)**	36.2 ± 22.0	40.9 ± 24.3	0.138
**Creatine (mg/dL)**	1.04 ± 0.90	1.03 ± 0.51	0.938
**Sodium (mmol/L)**	138.7 ± 2.81	138.1 ± 3.68	0.218
**Potassium (mmol/L)**	4.22 ± 0.55	4.45 ± 0.58	0.004 *
**ALT (U/L)**	27.7 ± 48.5	34.2 ± 58.1	0.375
**AST (U/L)**	27.1 ± 28.6	53.9 ± 88.7	0.003 *
**Troponin I (ng/L)**			
<100 [n (%)]	88 (79.3)	23 (20.7)	0.000 *
>100 [n (%)]	18 (17.8)	83 (82.2)	

* Significance between groups: *p* < 0.05. Analysis by independent samples *t*-test, mean ± standard deviation (SD), and chi-square (χ2), n (%). ACS: acute coronary syndrome, n: number, BUN: Blood Urea Nitrogen, ALT: alanine aminotransferase, AST: aspartate aminotransferase.

**Table 3:** Distribution of MTHFR 677 C/T and DNMT-1 +32204 A/G genotypes and allele frequencies in groups.

Polymorphic Site	Genotype/Allele	Control (n = 106)	ACS (n = 106)	OR (95% CI)	*p*–Value
**MTHFR** **677 C/T** **(rs1801133)**	**CC**	33 (36.7%)	57 (63.3%)	7.34 (2.28–23.6)	0.000 *
	**CT**	56 (55.4%)	45 (44.6%)	3.42 (1.07–10.8)	0.03 *
	**TT**	17 (81.0%)	4 (19.0%)	1 (reference)	
	χ2 = 15.6 df = 2 *p* = 0.000 *				
	**C**	122 (43.4%)	159 (56.6%)	2.21 (1.46–3.35)	0.000 *
	**T**	90 (62.9%)	53 (37.1%)	1 (reference)	
	χ2 = 14.4 df = 1 *p* = 0.000 *				
**DNMT-1 +32204 A/G** **(rs2228611)**	**GG**	33 (57.9%)	24 (42.1%)	1 (reference)	
	**GA**	48 (47.5%)	53 (52.5%)	1.52 (0.79–2.92)	0.210
	**AA**	25 (46.3%)	29 (53.7%)	1.60 (0.75–3.38)	0.221
	**χ2 = 1.96 df = 2 *p* = 0.374**				
	**G**	114 (53.0%)	101 (47.0%)	1 (reference)	
	**A**	98 (46.9%)	111 (53.1%)	1.28 (0.87–1.87)	0.207
	**χ2 = 1.59 df = 1 *p* = 0.207**				

* significance between groups: *p* < 0.05. Analysis by chi-square (χ^2^), n (%). MTHFR: methylenetetrahydrofolate reductase, DNMT-1: DNA methyltransferase 1, OR (95% CI): odds ratio 95% confidence intervals.

**Table 4:** Relation between MTHFR 677 C/T and DNMT-1 +32204 A/G genotypes and the characteristics of the control group.

Characteristic	MTHFR 677 C/T Genotypes			*p*-Value	DNMT-1 +32204 A/G Genotypes			*p*-Value
	CC	CT	TT		GG	GA	AA	
**Age**	51.6 ± 20.2	50.4 ± 18.2	51.8 ± 20.7	0.944	47.3 ± 17.2	51.7 ± 17.2	54.8 ± 24.2	0.322
**Sex**								
Female [n (%)]	10 (30.3)	25 (44.6)	7 (41.2)	0.406	15 (45.5)	16 (33.3)	11 (44.0)	0.481
Male [n (%)]	23 (69.7)	31 (55.4)	10 (58.8)		18 (54.5)	32 (66.7)	14 (56.0)	
**Glucose (mg/dL)**	121.7 ± 71.7	121.9 ± 51.2	123.4 ± 80.1	0.996	112.2 ± 47.1	130.8 ± 70.9	118.4 ± 63.4	0.401
**BUN (mg/dL)**	37.0 ± 16.7	36.6 ± 26.7	33.0 ± 13.2	0.815	29.9 ± 13.0	38.8 ± 26.2	39.4 ± 21.8	0.145
**Creatine (mg/dL)**	1.00 ± 0.36	1.10 ± 1.20	0.93 ± 0.34	0.749	0.87 ± 0.30	1.18 ± 1.28	1.01 ± 0.40	0.328
**Sodium (mmol/L)**	139.1± 2.16	138.5 ± 3.25	138.7 ± 2.44	0.625	138.6 ± 2.17	138.4 ± 2.53	139.4 ± 3.89	0.354
**Potassium (mmol/L)**	4.29 ± 0.46	4.22 ± 0.62	4.07 ± 0.44	0.423	4.22 ± 0.44	4.19 ± 0.49	4.29 ± 0.76	0.766
**ALT (U/L)**	22.9 ± 12.6	32.2 ± 65.1	22.1 ± 20.6	0.602	33.7 ± 62.9	29.1 ± 49.3	16.9 ± 9.49	0.416
**AST (U/L)**	24.3 ± 9.72	30.5 ± 38.3	21.1 ± 8.57	0.401	31.7 ± 34.5	27.2 ± 31.1	20.7 ± 6.45	0.357
**Troponin I (ng/L)**								
<100 [n (%)]	27 (81.8)	46 (82.1)	15 (88.2)	0.822	28 (84.8)	41 (85.4)	19 (76.0)	0.563
>100 [n (%)]	6 (18.2)	10 (17.9)	2 (11.8)		5 (15.2)	7 (14.6)	6 (24.0)	

Analysis by one-way ANOVA, mean ± standard deviation (SD), and chi-square (χ^2^), n (%). MTHFR: methylenetetrahydrofolate reductase, DNMT-1: DNA methyltransferase 1; n: number; BUN: Blood Urea Nitrogen, ALT: alanine aminotransferase, AST: aspartate aminotransferase.

**Table 5:** Relation between MTHFR 677 C/T and DNMT-1 +32204 A/G genotypes and the characteristics of the ACS group.

Characteristic	MTHFR 677 C/T Genotypes			*p*-Value	DNMT-1 +32204 A/G Genotypes			*p*-Value
	CC	CT	TT		GG	GA	AA	
**Age**	62.1 ± 12.8	61.4 ± 15.6	54.2 ± 16.8	0.560	59.0 ± 13.6	63.5 ± 12.8	60.1 ± 16.7	0.348
**Sex**								
Female [n (%)]	20 (35.1)	19 (42.2)	0 (0)	0.226	7 (29.2)	24 (45.3)	8 (27.6)	0.192
Male [n (%)]	37 (64.9)	26 (57.8)	4 (100)		17 (70.8)	29 (54.7)	21 (72.4)	
**Glucose (mg/dL)**	146.4 ± 56.2	168.7 ± 92.3	207.0 ± 122.5	0.150	144.7 ± 68.9	164.1 ± 87.0	158.3 ± 62.9	0.597
**BUN (mg/dL)**	38.1 ± 23.6	45.0 ± 25.8	35.1 ± 4.59	0.322	39.8 ± 26.0	41.7 ± 19.4	40.5 ± 30.9	0.945
**Creatine (mg/dL)**	0.99 ± 0.37	1.10 ± 0.66	1.04 ± 0.23	0.564	1.06 ± 0.65	1.05 ± 0.50	0.99 ± 0.40	0.835
**Sodium (mmol/L)**	138.3 ± 3.01	137.6 ± 4.26	141.0 ± 4.83	0.193	137.8 ± 2.98	138.4 ± 4.09	137.9 ± 3.47	0.744
**Potassium (mmol/L)**	4.36 ± 0.55	4.57 ± 0.61	4.42 ± 0.51	0207	4.42 ± 0.58	4.52 ± 0.55	4.35 ± 0.65	0.462
**ALT (U/L)**	29.6 ± 48.9	38.8 ± 69.9	47.2 ± 30.4	0.661	23.7 ± 9.91	43.7 ± 80.1	25.6 ± 18.4	0.246
**AST (U/L)**	42.6 ± 53.2	67.0 ± 121.2	67.7 ± 43.2	0.373	43.5 ± 35.0	119.2 ± 16.3	37.1 ± 6.90	0.256
**Troponin I (ng/L)**								
<100 [n (%)]	18 (31.6)	5 (11.1)	0 (0)	0.025 *	5 (20.8)	11 (20.8)	7 (24.1)	0.932
>100 [n (%)]	39 (68.4)	40 (88.9)	83 (100)		19 (79.2)	42 (79.2)	22 (75.9)	
**Diagnosis**								
STEMI [n (%)]	11 (19.3)	7 (15.6)	1 (25)	0.321	4 (13.3)	9 (16.9)	6 (20.7)	0.434
NSTEMI [n (%)]	35 (61.4)	35 (77.8)	2 (50)		13 (63.3)	39 (73.6)	20 (69.0)	
Unstable angina [n (%)]	11 (19.3)	3 (6.6)	1 (25)		7 (23.6)	5 (9.5)	3 (10.3)	

* significance between groups: *p* < 0.05. Analysis by one-way ANOVA, mean ± standard deviation (SD), and chi-square (χ2), n (%). MTHFR: methylenetetrahydrofolate reductase, DNMT-1: DNA methyltransferase 1, ACS: acute coronary syndrome, n: number, BUN: Blood Urea Nitrogen, ALT: alanine aminotransferase, AST: aspartate aminotransferase, STEMI: ST-elevation myocardial infarction, NSTEMI: non-ST elevation myocardial infarction.

## Data Availability

All data generated or analyzed during this study are included in this published article.

## Abbreviations

ACS	Acute coronary syndrome
MTHFR	Methylenetetrahydrofolate reductase
DNMT-1	Deoxyribonucleic acid methyltransferase-1
PCR	Polymerase chain reaction
RFLP	Restriction fragment length polymorphism
DNA	Deoxyribonucleic acid
OR	Odds ratio
CI	Confidence intervals
SCD	Sudden cardiac death
CAD	Coronary artery disease
ECG	Electrocardiogram
EDTA	Ethylenediaminetetraacetic acid
AST	Aspartate aminotransferase
ALT	Alanine aminotransferase
STE	ST-segment elevation
NSTEMI	Non-ST elevation myocardial infarction
STEMI	ST-elevation myocardial infarction
ESC	European Society of Cardiology
AHA	American Heart Association
ACC	American College of Cardiology
cTn	Cardiac troponin
SD	Standard deviation
ANOVA	One-way analysis of variance
χ^2^	Chi-square
